# A bibliometric analysis of publications in *Ambio* in the last four decades

**DOI:** 10.1007/s11356-021-14796-4

**Published:** 2021-07-24

**Authors:** Yuh-Shan Ho, Andrea Gatto

**Affiliations:** 1 Trend Research Centre, Asia University, No. 500, Lioufeng Road, Wufeng, Taichung, 41354 Taiwan; 2 Wenzhou-Kean University, Wenzhou, 325060 Zhejiang Province China; 3 Natural Resources Institute, University of Greenwich, Central Avenue, Chatham Maritime, Kent, ME4 4TB UK; 4 Centre for Studies on Europe, Azerbaijan State University of Economics (UNEC), Baku, Azerbaijan

**Keywords:** Bibliometrics, SCI-EXPANDED, *TC*_year_, *C*_year_, *CPP*_year_, Journal impact factor contributing papers, Journal impact factor contributing indicators, Journal impact factor contributors, *Ambio*, C0, I2, Q0

## Abstract

*Ambio* is a leading journal in environmental science and policy, sustainable development, and human-environment interactions. The paper at hand aims to run a bibliometric analysis to inspect the main publications features of *Ambio* in Science Citation Index Expanded SCI-EXPANDED. For this scope, a bibliometric survey has been carried out to investigate the journal’s historic characteristics in the Web of Science (WoS) categories of environmental sciences and environmental engineering for *Ambio* from 1980 to 2019. These are the categories for which the journal has been indexed throughout the indexed time frame. The paper proposes technical and methodological innovations, including improvements in the methods and original characteristics analyzed. Documents published in *Ambio* were searched out from SCI-EXPANDED. Six publication indicators were applied to evaluate the publication performance of countries, institutes, and authors. Three citation indicators were used to compare publications. As a parameter, the journal impact factor contributor was applied to compare the most frequently cited publications. The journal impact factor contributing publications were also discussed. Results show that Sweden ranked top in six publication indicators and that the top three productive institutes were located in Sweden. A low percentage of productive authors emerged as a journal impact factor contributor. Similarly, a low relationship between the *IF* contributing publications and the highly cited publications was also found. Less than half of the top 100 highly cited publications in *Ambio* did not lie within the high impact in most the recent year of 2019. Three members of the advisory board in *Ambio* were the main productive authors. T.V. Callaghan contributed to most of the publications while papers published by J. Rockstrom as first and corresponding author contributed the most to the journal impact factor. An article authored by Steffen et al. (2007) scored the highest total citations in 2019.

## Introduction

*Ambio* is a primary source for environmental scientists and policy analysts, as well as for scholars interested in sustainable development, ecosystem functioning and human-environment interactions. The journal is published by The Royal Swedish Academy of Sciences and was founded in 1971.

*Ambio* has been indexed in the Science Citation Index Expanded (SCI-EXPANDED) in 1977 and classified in the Web of Science (WoS) categories of environmental engineering and environmental sciences, performing an impact factor of 4.778 in 2019. Currently, *Ambio* is indexed in four WoS categories: ecology; environmental chemistry; geography, planning and development; medicine.

Sustainable development, sustainability studies and, more broadly, ecological and resource policy are ascending multidimensional disciplines that deserve devoted research, classification and data (Gatto and Drago 2021; Farrukh et al. 2020; Gatto 2020). Bibliometrics can be a valuable method to map and disentangle existing social science research in applied sciences (Ho and Gatto, 2020) and economics (Drago and Agizza 2019; Drago and Aliberti 2018). Additionally, diverse bibliometric techniques have been employed to wrap up and document complex phenomena and research progress in sustainable development and environmental policy (Gatto et al. 2020; Gatto and Drago 2020; Drago and Aliberti, 2018). Indeed, bibliometrics contemplates the use of diverse techniques, procedures and software to examine a wide range of publication characteristics. A large number of bibliometric analyses has been focusing on capturing the salient information sorting from specific journals' publications (Donthu et al. 2021; Baker et al. 2021). These pieces of evidence addressed and motivated this work’s rationale—opting for bibliometric methods that allow portraying past scholarship on the sketched issues, providing a comprehensive quantitative picture of a journal’s outputs within the social, economic and environmental sciences.

In the original two categories for which *Ambio* was first indexed—environmental engineering and environmental sciences—previous bibliometric analyses focusing on selected journals have been presented to study the journals’ development and characteristics. This is the case for the *Journal of Environmental Engineering and Landscape Management* (Xu et al. 2019), the *Indoor Air Quality* (Pierpaoli and Ruello 2018), the *Resources Conservation and Recycling* (Ji et al. 2018), the *Polish Journal of Environmental Studies* (Chuang et al. 2012), *Water Research* (Wang et al. 2010), *Industrial Health* (Smith and Leggat 2009), and the *Archives of Environmental Health* (Smith 2009). Five publication indicators were applied to evaluate the publication performance of countries and institutes in *Water Research* (Wang et al. 2010). Two citation indicators were presented for highly cited articles and the most impactful articles in the most recent year (Chuang et al. 2012). Citations per publication were used as a variable to compare the impact of document types and countries in the *Polish Journal of Environmental Studies* (Chuang et al. 2012). Recently, the journal impact factor contributing publications and authors were discussed (Ho 2019). It was pointed out that highly cited papers in a journal might not contribute to the journal impact factor.

In this study, documents published in *Ambio* from 1980 to 2019 were analyzed. Highly cited papers and authors, as well as the journal impact factor contributing publications and contributors, were examined and discussed. The aim was to explore the dynamics of the most salient publication for the target journal over time. The work also presents methodological innovations and technical improvements to the existing bibliometrics literature—i.e., upgraded procedural inputs and novel analysis proposals.

The remainder of the paper follows this organization: the “Methodology” section inspects the methodology used—i.e., the bibliometric analysis. The “Results and discussion” section presents the outcoming results from the analysis run. In this section, the characteristics of published papers; the characteristics of journal impact factor; the trends of publication numbers and citations per publication; countries, institutions, and authors of the published papers; highly cited papers; and words in title and author keywords are examined. This section also furnishes elements for discussion. Lastly, the “Conclusion” section wraps up the paper’s conclusions and presents research limitations and prospects.

## Methodology

The documents used in this study were derived from the SCI-EXPANDED of the WoS Core Collection, Clarivate Analytics. The searching keyword phrase “*Ambio*” was searched as a publication name based on SCI-EXPANDED. The study considered the largest available time frame—i.e., from 1980 to 2019 (updated on December 2, 2020). All document information from SCI-EXPANDED and each year’s citation times for every publication sorting from the Web of Science Core Collection was checked and downloaded into Microsoft Excel 2016. Additional coding was manually performed.

The SCI-EXPANDED database was originally designed for researchers to find documents, but it cannot be directly used for bibliometric research (Ho 2018). Therefore, when using the SCI-EXPANDED database, data should be treated before bibliometric processing. The chosen database has been analyzed through Excel. Data were controlled, cleaned and classified. Data were inputted and treated through diverse analysis techniques. To this end, a large number of codes have been exploited to optimize the word filtering and selection, ensuring methodological soundness and overall correctness of this exercise. This includes “proper” and “lower” functions, which have been used to standardize the words—to ensure that similarly written strings were not left behind. This way, when the same words were spelt differently, these could be included in the analysis without misleading overlaps. Another key code was “match,” which allowed the possibility to capture one paper published in different countries, taking into account all of them. At the same time, this step facilitated the avoidance of overlappings in the numbers of institutes, countries and additional variables. Further implemented codes included “vlookup” and “concatenate,” which were used to guarantee proper network analysis outputs.

The performed research has been undertaken through a brand-new set of feature analysis that has been completed by the traditional key publication features explorations. This includes taking into account: (i) selected characteristics percentages and ranks together; (ii) independent studies; (iii) international collaborations; (iv) most performative first authors and corresponding authors; (v) characteristics of the most productive countries; (vi) characteristics of the most productive institutions; (viii) most recurrent words within the publication’s title.

The collaboration type, country and institution were determined by the authors’ affiliation. In the SCI-EXPANDED database, the corresponding author is designated as the “reprint author”. This study uses the term “corresponding author” instead. In a single author publication where the authorship is unspecified, the single author was considered both as first author and corresponding author. Similarly, in a singly institutional publication, the institution is classified as the first-author institution and the corresponding-author institution. In multi-corresponding-author articles, only the last corresponding author, institute, and country were considered (see Ho 2019).

The affiliations that originated in England, Scotland, Northern Ireland and Wales were reclassified as UK (United Kingdom). For publications that occurred before 1997, Hong Kong affiliates were included in China. The affiliations in Trinid & Tabago were included in Trinidad Tobago. The affiliates of Fed Rep Ger (Federal Republic of Germany) and Ger Dem Rep (German Democratic Republic) were also included in Germany. France includes the affiliation of French Guiana. The affiliations in Greenland were included in Denmark. The affiliation of Czechoslovakia was checked and reclassified as the Czech Republic. The affiliation in the USSR (Union of Soviet Socialist Republics) were checked and reclassified as in Russia and Estonia—depending on the institution’s nationality. The members of W Ind Assoc St (West Indies Associated States) were reclassified as St Lucia (Saint Lucia) affiliates. Similarly, Geol Survey Czechoslovakia (Geological Survey of Czechoslovakia) was checked and reclassified as Czech Geol Survey (Czech Geological Survey). Acad Sci USSR (the Academy of Sciences of the Soviet Union) was checked and reclassified as Russian Acad Sci (the Russian Academy of Sciences). The same applied to the Acad Med Sci USSR (the USSR Academy of Medical Sciences)—it was checked and reclassified as Russian Acad Med Sci (the Russian Academy of Medical Sciences). Publications by Stockholm Univ and Univ Stockholm were also reclassified as pertaining to Stockholm Univ (Stockholm University in Sweden).

## Results and discussion

### Characteristics of document type

From 1980 to 2019, *Ambio* published 4154 documents in 10 Web of Science document types.

Table 1 shows the characteristics of these 10 document types, including 3263 articles (79% of 4154 documents) with a number of authors per publication (*APP*) of 3.3. It is worth noting that the documents in the Web of Science Core Collection can be divided into two document types. Among those, 137 documents are classified as conference papers and articles. Therefore, the total percentage is higher than 100%.

**Table 1:** Citations and authors according to document type

Document type	TP	%	TP*	AU	APP	*TC*_2019_	*CPP*_2019_
Article	3263	79	3256	10,853	3.3	94,489	29
Editorial material	601	14	577	1108	1.9	2694	4.5
Review	142	3.4	142	820	5.8	4701	33
Proceedings paper	137	3.3	137	454	3.3	8848	65
Note	118	2.8	112	158	1.4	734	6.2
Correction	12	0.29	12	40	3.3	3	0.25
Letter	9	0.22	9	20	2.2	59	6.6
Addition correction	7	0.17	7	8	1.1	1	0.14
Biographical-item	1	0.024	1	3	3.0	0	0
Discussion	1	0.024	1	7	7.0	7	7.0

*TP*, number of papers; TP*, number of papers with author information in SCI-EXPANDED; *AU*, number of authors; *APP*, number of authors per publication; *TC*_2019_, the total number of citations from Web of Science Core Collection since publication to the end of 2019; *CPP*_2019_, number of citations (*TC*_2019_) per publication (*TP*)

Document type of discussions with one document had the highest *APP* of 7.0 followed by reviews with 5.8. The average number of authors per publication in the journal was 3.2, and the maximum number of authors was 53.

The article entitled “Multi-decadal changes in tundra environments and ecosystems: Synthesis of the international polar year-back to the future project (IPY-BTF)” (Callaghan et al. 2011) was published by 53 authors. The co-authors were affiliated to the Royal Swedish Academy of Sciences in Sweden, the University of Sheffield in the UK, and the University of Texas at El Paso in the USA. The *CPP*_2019_ of the proceedings paper was much higher than other document types, at 65. Reviews had similar *CPP*_2019_ than articles. A review entitled “Coupled human and natural systems” (Liu et al. 2007) was co-authored by 15 authors from the USA, Sweden, and China. This paper was the most frequently cited review in *Ambio* with a *CPP*_2019_ of 408.

According to the Web of Science’s definition,^1^ the journal impact factor (*IF*_year_) is defined as in the Journal Citation Reports (JCR) year—i.e., the average number of citations of a journal article published in the past two years. The denominator includes document types of articles or reviews. Some categories of documents that are not normally cited, for example, letters, editorial materials, and other document types are not included in the denominator of the impact factor. Due to the definition of the journal impact factor, only the following document types were considered for further analysis: articles, reviews, and proceedings papers.

### Characteristics of the journal impact factor (*IF*)

According to the 2019 Journal Citation Reports (JCR), JCR used 178 Web of Science categories in SCI-EXPANDED to index 9381 journals. The journal impact factor (*IF*) is defined as the sum of all the citations coming from articles published by the journal in the previous two years in the selected JCR year, divided by the total number of academic articles published by the journal (including articles, reviews and proceedings papers). Periodicals of the first two years have observed the following: (https://incites.help.clarivate.com/Content/Indicators-Handbook/ih-journal-impact-factor.htm?Highlight=impact%20factor).

The journal impact factor has the following formula:

${IF}_{Year}=\frac{C_{year-2}+{C}_{year-1}}{TP_{Year-2}+{TP}_{Year-1}}$where *IF*_year_ is the journal impact factor in a specific JCR year, *C*_year-2_: citations from JCR year to items in “year - 2,” *C*_year-1_: citations from JCR year to items in “year - 1,” *TP*_year-2_: citable items in “year - 2,” *TP*_year-1_: citable items in “year - 1.” *CN*: the journal impact factor contributing indicator (*C*_year-1_ + *C*_year-2_) that means the citation number of *C*_year-1_ + *C*_year-2_. The denominator is made of three document types such as reviews, articles, and proceedings papers. A research product pertaining to any other document type is excluded from the denominator.

**Fig. 1:**
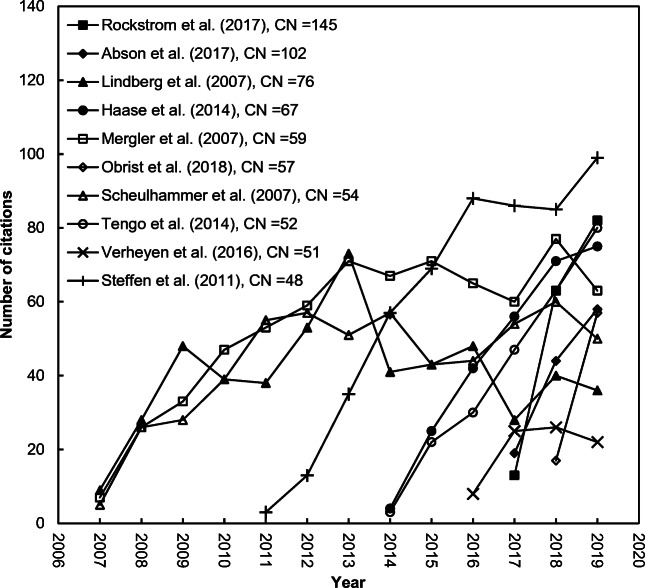
Citation histories of the top ten journal impact factor contributing papers

The top 20 journal impact factor (*IF*) contributing papers were listed in Table 2. 70%, 20%, and 10% of them were published in the 2010s, 2000s, and 1990s, respectively. 75% and 70% of the top 20 most *IF* contributing papers were ranked top 20 in terms of *CN* (*C*_year-2_ + *C*_year-1_), respectively. However, only 25% and 50% of them were ranked as top 20 in terms of *TC*_2019_ and *C*_2019_, respectively. Only five of the top 20 most contributing papers for the journal impact factor (*IF*) contributing papers including four articles by Mergler et al. (2007), Scheulhammer et al. (2007), Steffen et al. (2011), and Lindberg et al. (2007) and a review by Madronich et al. (1995), ranked within the top 20 in total citations with *TC*_2019_ of 699, 569, 535, 524, and 322, respectively. However, 10 of the top 20 highest *IF* contributing papers—including two in 2007, one in 2011, three in 2014, one in 2016, two in 2017, and one in 2018—ranked in the top 20 with a *C*_2019_ ≥ 43 as the most impactful papers in the most the recent year—2019. Furthermore, 33% and 52% of the top 100 *IF* contributing papers in terms of *CN* were found in the top 100 papers in terms of *TC*_2019_ and *C*_2019_, respectively; and only 23% of the top 100 papers in terms of *CN* were found in both the top 100 *C*_2019_ and *TC*_2019_. It can be concluded that the impact factor of a journal is used to evaluate a journal’s relative importance, especially when compared to others in the same field but it is not an appropriate metric when compared to individual research performance. It was also reported in the Web of Science that the journal impact factor (*IF*) is a paper-level metric. It does not apply to individual papers or subgroups of papers that appeared in the publication. Additionally, it does not apply to authors of papers, research groups, institutions, or universities.^2^ Figure 1 shows the citation histories of the top ten highest journal impact factor contributing papers. Three papers are still keeping an increased trend of citations: Haase et al. (2014), Tengo et al. (2014), and Steffen et al. (2011).

**Table 2:** Top 20 journal impact factor contributing papers in *Ambio*

Title (reference)	Rank (*CN*)	Rank (*C*_year-2_)	Rank (*C*_year-1_)	Rank (*TC*_2019_)	Rank (*C*_2019_)
Sustainable intensification of agriculture for human prosperity and global sustainability (Rockstrom et al. 2017)	1 (145)	1 (82)	1 (63)	87 (159)	5 (82)
Leverage points for sustainability transformation (Abson et al. 2017)	2 (102)	2 (58)	3 (44)	150 (121)	9 (58)
A synthesis of progress and uncertainties in attributing the sources of mercury in deposition (Lindberg et al. 2007)	3 (76)	3 (48)	5 (28)	9 (524)	26 (36)
Ecosystem services in urban landscapes: Practical applications and governance implications (Haase et al. 2014)	4 (67)	4 (42)	8 (25)	24 (273)	7 (75)
Methylmercury exposure and health effects in humans: A worldwide concern (Mergler et al. 2007)	5 (59)	6 (33)	6 (26)	5 (699)	8 (63)
A review of global environmental mercury processes in response to human and natural perturbations: Changes of emissions, climate, and land use (Obrist et al. 2018)	6 (57)	2020 (0)	2 (57)	299 (74)	10 (57)
Effects of environmental methylmercury on the health of wild birds, mammals, and fish (Scheulhammer et al. 2007)	7 (54)	11 (28)	6 (26)	7 (569)	14 (50)
Connecting diverse knowledge systems for enhanced ecosystem governance: The multiple evidence base approach (Tengo et al. 2014)	8 (52)	8 (30)	12 (22)	35 (245)	6 (80)
Contributions of a global network of tree diversity experiments to sustainable forest plantations (Verheyen et al. 2016)	9 (51)	15 (26)	8 (25)	266 (81)	54 (22)
The anthropocene: From global change to planetary stewardship (Steffen et al. 2011)	10 (48)	5 (35)	25 (13)	8 (535)	3 (99)
When is a forest a forest? Forest concepts and definitions in the era of forest and landscape restoration (Chazdon et al. 2016)	11 (46)	13 (27)	14 (19)	218 (93)	17 (43)
Changes in ultraviolet-radiation reaching the earth’s surface (Madronich et al. 1995)	12 (44)	7 (32)	30 (12)	17 (322)	417 (5)
Ecological and socioeconomic impacts of 1998 coral mortality in the Indian Ocean: An ENSO impact and a warning of future change? (Wilkinson et al. 1999)	12 (44)	8 (30)	22 (14)	30 (256)	287 (7)
Reconnecting cities to the biosphere: Stewardship of green infrastructure and urban ecosystem services (Andersson et al. 2014)	14 (42)	10 (29)	25 (13)	69 (176)	13 (53)
Phosphorus management in Europe in a changing world (Schoumans et al. 2015)	14 (42)	36 (17)	8 (25)	199 (100)	31 (31)
Future agriculture with minimized phosphorus losses to waters: Research needs and direction (Sharpley et al. 2015)	16 (40)	22 (21)	14 (19)	191 (102)	33 (30)
Changing Arctic snow cover: A review of recent developments and assessment of future needs for observations, modelling, and impacts (Bokhorst et al. 2016)	17 (39)	11 (28)	39 (11)	425 (57)	101 (14)
Societal transformation in response to global environmental change: A review of emerging concepts (Feola 2015)	18 (36)	25 (20)	18 (16)	181 (104)	24 (37)
Contribution of ecosystem services to air quality and climate change mitigation policies: The case of urban forests in Barcelona, Spain (Baró et al. 2014)	18 (36)	16 (25)	39 (11)	159 (116)	27 (34)
Recovery of mercury-contaminated fisheries (Munthe et al. 2007)	20 (35)	36 (17)	16 (18)	48 (221)	154 (11)

*CN*, journal impact factor contributing indicator (*C*_year-1_ + *C*_year-2_)

*C*_year-2_, number of citations from JCR year to papers in “year - 2”

*C*_year-1_, number of citations from JCR year to papers in “year - 1”

*TC*_2019_, total number of citations from Web of Science Core Collection since publication year to the end of 2019

*C*_2019_, total number of citations from Web of Science Core Collection in 2019

**Fig. 2:**
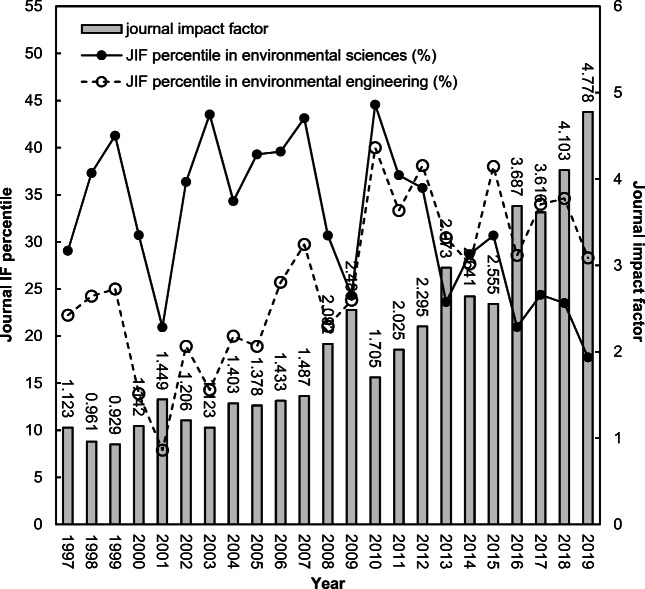
Rankings of *Ambio* by journal impact factor in Web of Science categories of environmental sciences and environmental engineering from 1997 to 2019

As previously stated, *Ambio* has been classified in the Web of Science categories of environmental sciences and environmental engineering from its very first indexing. Figure 2 shows its *IF* and ranking within the subject categories from 1997 to 2019. Within the years, the journal impact factor (*IF*) fluctuated and slightly increased in the long run, reaching a minimum of 0.929 in 1999 to a maximum of 4.778 in 2019. The ranking of *Ambio* in the Web of Science subject categories of environmental sciences and environmental engineering were not improved, especially in the last five years. The journal had a higher ranking in the category of environmental engineering with respect to environmental sciences since 2012. In 2014, notable changes occurred in *Ambio*’s policies, including the enclosure of six major new features (Söderström 2014).

It is clear from Fig. 2 that the journal impact factor increased after that change. The time required to accumulate the citations affects the impact (Garfield 1999). The size of the scientific community that a journal serves also affects the journal’s impact (Garfield 1999). How quickly recent publications are cited is another important factor that can affect *IF* (Garfield 1999). In general, citations per publication for papers in a journal would have a sharp increase after publication and would reach a peak in a specific year (Fu and Ho 2015; Monge-Nájera and Ho 2016).

Figure 2 shows the citations per publication for each year of paper life. The peak year of citations per publication with 2.5 was found to be in the 3rd full year since its publication. That differed from *Revista de Biología Tropical* (*IF*_2019_ = 0.446) with the peak year in the 7th year (Monge-Nájera and Ho 2016); the *Journal of Membrane Science* (*IF*_2019_ = 7.183) in the 4th year (Fu and Ho 2015); and the *Journal of Orthopaedic Research* (*IF*_2019_ = 2.728) in the 5th year (Ho 2019). A special case not showing a peak was reported—unlike previous findings. However, it shows an increasing trend without a peak after the 9th year for the *Polish Journal of Environmental Studies* (*IF*_2019_ = 1.383) (Chuang et al. 2012). Since the *IF* only considers citations within two years after publication, the *IF* of *Ambio* can be reputed as acceptable. It was pointed out that the *IF* is not an unbiased criterion for all journals, since peak year citations per publication of each journal can be different from each other (Chuang et al. 2012).

### Trends of publication numbers and citations per publication

**Fig. 3:**
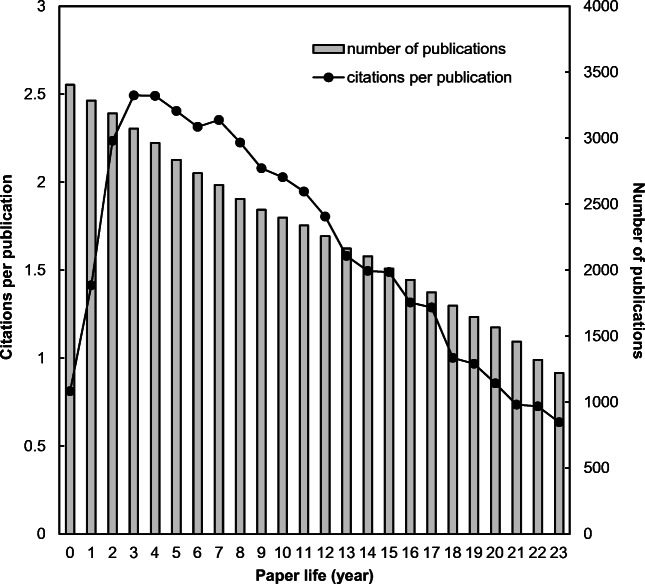
Trend of citations per publication

The trends of publication numbers show a journal’s development, whereas the citations per publication variable provide information about the impact of a journal in the research world. The trends of the number of annual articles (*TP*) and their citations per publication (*CPP*_2019_ = *TC*_2019_/*TP*) have been proposed to discover the development of a journal (Fu and Ho 2015; Monge-Nájera and Ho 2016; Ho 2019). Amidst the 3405 papers including 3263 articles with *CPP*_2019_ of 29 and 142 reviews *CPP*_2019_ of 33 published in *Ambio* were analyzed. The trend of citations per publication is sketched in Fig. 3.

**Fig. 4:**
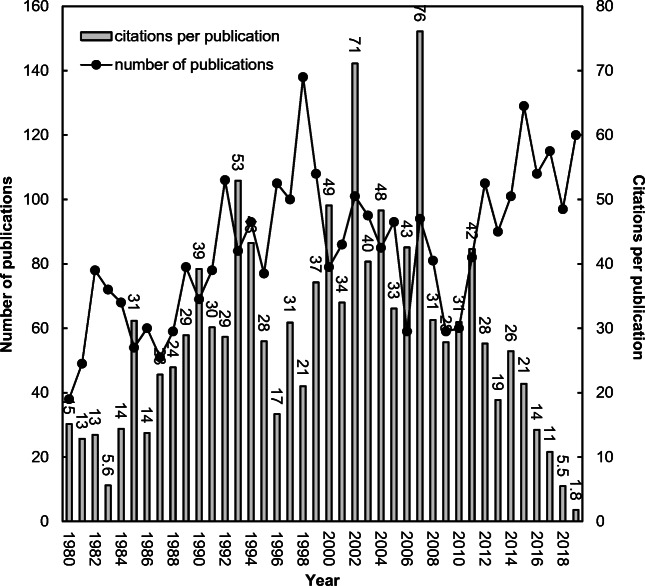
Number of papers and citations per publication versus year for *Ambio*

The number of annual papers fluctuated with an increase in the last decade. An average of 85 papers was found from 38 in 1980 to 138 in 1998 (Fig. 4). In 2007, 94 papers had the highest *CPP*_2019_ of 76 followed by 71 in 2002. Based on Fig. 4, it roughly takes a decade to the *CPP*s for reaching a plateau. Similarly, results for the *Revista de Biología Tropical* (Monge-Nájera and Ho 2016), the *Journal of Membrane Science* (Fu and Ho 2015), and the *Journal of Orthopaedic Research* (Ho 2019) also took about one decade to reach a plateau.

### Countries, institutions, and authors of the published papers

Excluding 144 papers without the author’s affiliation information on SCI-EXPANDED, the remaining 3261 papers originated from 126 different countries. Among those papers, 2242 (69% of the 3261 papers) were single country papers from 87 countries, while 1019 (31%) were internationally collaborative papers from 117 countries. Six publication indicators: number total of papers (*TP*), country independent papers (*IP*), internationally collaborative papers (*CP*), first-author papers (*FP*), corresponding-author papers (*RP*), and single-author papers (*SP*) have been applied as criteria to compare the publication’s performance of countries and institutes for a research topic (Vega-Arce et al. 2019).

Table 3 shows the top 10 most contributing countries with the six publication indicators presented. Six European countries including four countries in Scandinavia, two in America, one in Oceania, and one in Asia were ranked on the top 10 of total papers. The most productive African country was Tanzania with 56 papers (ranked 19th). Sweden dominated in *Ambio*, ranking as the first in all six publication indicators with a *TP* of 1080 papers (33% of 3261 papers), an *IP* of 639 papers (29% of 2242 country independent papers), a *CP* of 441 papers (43% of 1019 internationally collaborative papers), an *FP* of 879 papers (27% of 3261 first-author papers), an *RP* of 872 papers (27% of 3227 corresponding-author papers), and an *SP* of 186 papers (22% of 838 single-author papers).

**Table 3:** Characteristics of the top 10 productive countries

Country	*TP*	*TP* *R* (%)	*IP* *R* (%)	*CP* *R* (%)	*FP* *R* (%)	*RP* *R* (%)	*SP* *R* (%)
Sweden	1080	1 (33)	1 (29)	1 (43)	1 (27)	1 (27)	1 (22)
USA	760	2 (23)	2 (18)	2 (36)	2 (16)	2 (16)	2 (18)
UK	372	3 (11)	3 (6.3)	3 (23)	3 (7.1)	3 (7.1)	3 (9.4)
Canada	232	4 (7.1)	4 (4.2)	4 (13)	4 (4.7)	4 (4.8)	4 (3.7)
Finland	216	5 (6.6)	5 (4.2)	6 (12)	5 (4.2)	5 (4.2)	6 (2.7)
Norway	198	6 (6.1)	7 (3.1)	5 (13)	6 (3.1)	6 (3.1)	14 (1.4)
Germany	175	7 (5.4)	10 (2.4)	7 (12)	10 (2.6)	10 (2.7)	9 (2.3)
Denmark	170	8 (5.2)	11 (2.4)	8 (11)	9 (2.9)	9 (2.9)	12 (1.7)
Australia	162	9 (5.0)	8 (2.8)	9 (10)	7 (3.0)	7 (3.0)	4 (3.7)
China	139	10 (4.3)	6 (3.3)	11 (6.5)	7 (3.0)	7 (3.0)	8 (2.4)

*TP*, total number of papers; *IP*, country independent papers; *CP*, internationally collaborative papers; *FP*, first-author papers; *RP*, corresponding-author papers; *SP*, single-author papers

Of the total 3261 papers with affiliation information in SCI-EXPANDED, 1447 papers (44% of 3261 papers) came from independent institutions with a *CPP*_2019_ of 25 and 1814 papers (56%) from inter-institutional collaborations with a *CPP*_2019_ of 34. The percentage of the inter-institutional collaboration rate of the *Ambio* (56%) was found higher than that of *J. Membr. Sci.* (38%) (Fu and Ho 2015), and *Pol. J. Environ. Stud.* (31%) (Chuang et al. 2012), but lower than that of *J. Orthop. Res.* (63%) (Ho 2019).

The characteristics of the top 10 productive institutions with the six publication indicators were listed in Table 4 (Vega-Arce et al. 2019). Seven of the top ten institutes were located in Sweden. The University of Helsinki in Finland ranked 4th, Aarhus University in Denmark ranked 8th and the University of Copenhagen in Denmark ranked 10th. Stockholm University in Sweden took the leading position in five of the six publication indicators with a *TP* of 308 papers (9.4% of 3261 papers), an *IP* of 68 papers (4.7% of 1447 institutional independent papers), a *CP* of 240 papers (13% of 1814 inter-institutionally collaborative papers), an *FP* of 159 papers (4.9% of 3261 first-author papers), and an *RP* of 157 papers (4.9% of 3227 corresponding-author papers) while the Swedish University of Agricultural Sciences in Sweden took the leading position with an *SP* of 27 papers (3.2% of 838 single-author papers).

**Table 4:** Characteristics of the top 10 productive institutes

Institute	*TP*	*TP R* (%)	*IP R* (%)	*CP R* (%)	*FP R* (%)	*RP R* (%)	*SP R* (%)
Stockholm University, Sweden	308	1 (9.4)	1 (4.7)	1 (13)	1 (4.9)	1 (4.9)	2 (3.0)
Swedish University of Agricultural Sciences, Sweden	225	2 (6.9)	2 (4.4)	2 (8.9)	2 (4.1)	2 (4.1)	1 (3.2)
Lund University, Sweden	88	3 (2.7)	9 (0.90)	3 (4.1)	6 (1.1)	6 (1.1)	31 (0.36)
University of Helsinki, Finland	83	4 (2.5)	3 (1.2)	5 (3.6)	6 (1.1)	6 (1.1)	8 (0.72)
Umea University, Sweden	80	5 (2.5)	10 (0.76)	4 (3.8)	4 (1.3)	4 (1.3)	12 (0.60)
University of Gothenburg, Sweden	79	6 (2.4)	7 (1.0)	6 (3.5)	3 (1.5)	3 (1.5)	3 (1.4)
Royal Swedish Academy of Sciences, Sweden	78	7 (2.4)	8 (1.0)	6 (3.5)	5 (1.2)	5 (1.2)	5 (1.2)
Aarhus University, Denmark	63	8 (1.9)	21 (0.41)	8 (3.1)	10 (0.86)	9 (0.87)	N/A
Uppsala University, Sweden	62	9 (1.9)	14 (0.62)	10 (2.9)	12 (0.83)	9 (0.87)	8 (0.72)
University of Copenhagen, Denmark	57	10 (1.7)	68 (0.21)	9 (3.0)	20 (0.49)	20 (0.5)	N/A

*TP*, total number of papers; *IP*, institute independent papers; *CP*, inter-institutionally collaborative papers; *FP*, first-author papers; *RP*, corresponding-author papers; *SP*, single-author papers; *CPP*_2019_, citations per publication (*CPP*_2019_ = *TC*_2019_/*TP*); *N/A*, not available

With the exception of seven anonymous papers , 3398 papers were published by 8431 authors including 2798 authors who published first-author papers, 2733 authors who published corresponding-author papers, and 834 authors who published single-author papers. The first author and the corresponding author are the most significant author positions (Gaeta 1999; Mattsson et al. 2011). Three bibliometric indicators such as total papers (*TP*), first-author papers (*FP*), corresponding-author papers (*RP*), and single-author papers (*SP*) were applied for the analysis of authors’ characteristics for papers in a research field.

Table 5 lists the top 17 productive authors with 13 papers in *Ambio*. T.V. Callaghan (*CPP*_2019_ = 43; *CN* = 247), who is a member of the advisory board in *Ambio*, affiliated to the Royal Swedish Academy of Sciences in Sweden, was the largest contributor with 40 papers including seven reviews and 33 articles. Callaghan also ranked top in first-author papers with 20 items and corresponding-author papers with 19 publications. F. Barnaby (*CPP*_2019_ = 2.0; *CN* = 11) ranked the top in single-author papers with 12. M. Johansson (*CPP*_2019_ = 46; *CN* = 163) with 27 papers ranked second in total papers and T.R. Christensen (*CPP*_2019_ = 34; *CN* = 109) with 22 papers ranked third, both of Johansson and Christensen were from Lund University in Sweden. C. Folke (*CPP*_2019_ = 227; *CN* = 278) with 20 papers from the Stockholm University in Sweden and T.D. Prowse (*CPP*_2019_ = 50; *CN* = 72) with 18 papers from the University of Victoria in Canada were also sitting in the advisory board of *Ambio*.

**Table 5:** Characteristics of the top 24 productive authors with *TP* ≥ 12

Author	Rank (*TP*)	Rank (*FP*)	Rank (*RP*)	Rank (*SP*)	*TC*_2019_	*CPP*_2019_	CN
T.V. Callaghan	1 (40)	1 (20)	1 (19)	72 (1)	1712	43	247
M. Johansson	2 (27)	52 (3)	49 (3)	N/A	1234	46	163
T.R. Christensen	3 (22)	52 (3)	49 (3)	N/A	737	34	109
C. Folke	4 (20)	13 (5)	25 (4)	N/A	4539	227	278
P. Angelstam	5 (18)	6 (8)	3 (9)	N/A	939	52	91
T.D. Prowse	5 (18)	4 (9)	3 (9)	N/A	903	50	72
A. Bergman	7 (17)	28 (4)	25 (4)	N/A	864	51	98
M. Olsson	7 (17)	13 (5)	12 (5)	N/A	882	52	84
O. Linden	7 (17)	28 (4)	25 (4)	23 (2)	685	40	87
L.O. Bjorn	10 (16)	109 (2)	107 (2)	N/A	1106	69	27
H.E.M. Meier	10 (16)	13 (5)	12 (5)	N/A	621	39	110
T. Elmqvist	10 (16)	367 (1)	364 (1)	N/A	2899	181	290
J.D. Reist	13 (14)	52 (3)	49 (3)	N/A	829	59	52
P. Okeefe	13 (14)	10 (6)	8 (6)	23 (2)	43	3.1	13
S. Lofgren	15 (13)	13 (5)	12 (5)	N/A	320	25	57
F. Barnaby	15 (13)	2 (13)	2 (12)	1 (12)	26	2.0	11
F. Wulff	15 (13)	10 (6)	8 (6)	N/A	1438	111	109
A. Bignert	18 (12)	N/A	N/A	N/A	485	40	57
M.C. Ohman	18 (12)	52 (3)	49 (3)	N/A	419	35	25
R. Axelsson	18 (12)	109 (2)	107 (2)	N/A	337	28	58
H. Laudon	18 (12)	109 (2)	107 (2)	N/A	249	21	97
W.F. Vincent	18 (12)	367 (1)	364 (1)	N/A	656	55	49
M. Elbakidze	18 (12)	52 (3)	49 (3)	N/A	337	28	58
S. Jonasson	18 (12)	N/A	N/A	N/A	478	40	37

*TP*, total number of papers; *FP*, first-author papers; *RP*, corresponding-author papers; *TC*_2019_, the total number of citations from Web of Science Core Collection since publication year to the end of 2019; *CPP*_2019_, citations per publication (*CPP*_2019_ = *TC*_2019_/*TP*); *CN*, *C*_year-1_ + *C*_year-2_; *C*_year-1_, number of citations from JCR year to papers in “year - 1”; *C*_year-2_, number of citations from JCR year to papers in “year - 2”; *N/A*, not available

E. Andersson (*CPP*_2019_ = 424; *CN* = 100) from Stockholm University in Sweden was the most productive associate editor in *Ambio*, and published seven articles. Comparing the top 24 most productive authors, an advisory board member—C. Folke—with one review and 19 articles, had the highest *CPP*_2019_ of 227, followed by T. Elmqvist (16 papers; *CPP*_2019_ = 181) and F. Wulff (13; *CPP*_2019_ = 111) from Stockholm University, respectively. T. Elmqvist from Stockholm University was the journal impact factor best contributor with the highest *CN* (*C*_year-1_ + *C*_year-2_) of 290, followed by C. Folke from the same university with a *CN* of 278, and T.V. Callaghan from Royal Swedish Academy of Sciences with a *CN* of 247. The most productive authors, C. Folke and T. Elmqvist also published the top largest number of papers falling within the 100 journal impact factor contributing papers with five contributions, respectively. A.D. Fox, C. Folke, J.N. Galloway, T.V. Callaghan, and W. Steffen published two first-author papers and corresponding-author papers in the top 100 journal impact factor contributing papers.

The article entitled “Sustainable intensification of agriculture for human prosperity and global sustainability” (Rockstrom et al. 2017) was the best contributing article in terms of journal impact factor in *Ambio* with a *CN* of 145 followed by the article entitled “Leverage points for sustainability transformation” (Abson et al. 2017) with a *CN* of 102. Furthermore, only 26% and 25% of the top 100 productive authors were ranked in the top 100 in terms of *CN* of their first author and corresponding author, respectively. J. Rockstrom from Stockholm University published five papers in *Ambio* including two first-author papers and two corresponding-author papers. Rockstrom had the highest *CN* of 150 for his first-author papers and corresponding-author papers, respectively. D.J. Abson from Leuphana University of Luneburg in Germany published two papers in *Ambio* including one first-authored paper and one corresponding-author paper. Abson had the second-highest *CN* of 102 for his first-author papers and corresponding-author papers, respectively.

### Highly cited papers

The total number of citations was obtained from the Web of Science Core Collection as the bibliometric indicator, *TC*_2019_. The main research fescues in a research topic might be reflected by highly cited articles. Highly cited papers in *JAMA-Journal of the American Medical Association* (Garfield 1997), *Water Research* (Wang et al. 2010), and *Polish Journal of Environmental Studies* (Chuang et al. 2012) were presented. Articles with a *TC*_year_ of 100 or more, were generally called highly cited articles (Ho and Gatto, 2020).

In *Ambio*, 202 (5.9%) of 3405 papers were highly cited papers including one classic article with a *TC*_2019_ of 1126. The 202 highly cited papers were published by 836 highly cited authors from 412 institutions in 45 countries. The USA had 86 highly cited papers (43% of 200 highly cited papers with affiliation information in SCI-EXPANDED) followed by Sweden (73 articles; 37%), Canada (28; 14%), the UK (24; 12%), and Germany (20; 10%). Stockholm University in Sweden published 32 of 202 highly cited papers (16% of the 202 Royal Swedish Academy of Sciences) followed by the Swedish University of Agricultural Sciences, Royal Swedish Academy of Sciences, and Lund University in Sweden with 10 papers. C. Folke published the most cited papers in the *Ambio* with 12 papers including the most four first-author and corresponding-author papers, respectively.

The top 20 most frequently cited papers were listed in Table 6. Five of the top 20 highly cited papers were published in 2002 and 2007, respectively. Six and 17 of the top 20 most frequently cited papers were ranked top 20 in terms of *CN* (*C*_year-2_ + *C*_year-1_), respectively while five and 10 of them were ranked top 20 in terms of *CN* and *C*_2019_, respectively. The five of the top 20 most frequently cited papers including four articles by Lindberg et al. (2007), Mergler et al. (2007), Scheulhammer et al. (2007), and Steffen et al. (2011) and a review by Madronich et al. (1995) ranked in the top 20 most *IF* contributing papers with a *CN* of 76, 59, 54, 48, and 44, respectively. Ten of the top 20 most frequently cited papers in *Ambio* were published by Steffen et al. (2007), Folke et al. (2002), Steffen et al. (2011), Cassman et al. (2002), Mergler et al. (2007), Liu et al. (2007), Scheulhammer et al. (2007), Costanza et al. (2008), Galloway and Cowling (2002), and Gadgil et al. (1993), and were also ranked the top 20 in *C*_2019_ as the most impactful papers in 2019. The only classic publication with a *TC*_2019_ of 1000 or more was an article entitled “The Anthropocene: Are humans now overwhelming the great forces of nature” (Steffen et al. 2007) published by Steffen et al., written by scholars whose affiliations were from Australia, Germany, and the USA. This most frequently cited article in *Ambio* was also the most impactful in most the recent year of 2019 with a *C*_2019_ of 144.

**Table 6:** Top 20 highly cited papers in *Ambio*

Rank (*TC*_2019_)	Rank (*C*_2019_)	Rank (*CN*)	Titles	References
1 (1126)	1 (144)	46 (24)	The Anthropocene: Are humans now overwhelming the great forces of nature	Steffen et al. (2007)
2 (991)	2 (121)	94 (16)	Resilience and sustainable development: Building adaptive capacity in a world of transformations	Folke et al. (2002)
3 (777)	3 (99)	197 (11)	Agroecosystems, nitrogen-use efficiency, and nitrogen management	Cassman et al. (2002)
4 (755)	16 (46)	37 (27)	Reactive nitrogen and the world: 200 years of change	Galloway and Cowling (2002)
5 (699)	8 (63)	5 (59)	Methylmercury exposure and health effects in humans: A worldwide concern	Mergler et al. (2007)
6 (649)	49 (24)	24 (32)	Global fractionation and cold condensation of low volatility organochlorine compounds in polar regions	Wania and Mackay (1993)
7 (569)	14 (50)	7 (54)	Effects of environmental methylmercury on the health of wild birds, mammals, and fish	Scheulhammer et al. (2007)
8 (535)	3 (99)	10 (48)	The anthropocene: From global change to planetary stewardship	Steffen et al. (2011)
9 (524)	26 (36)	3 (76)	A synthesis of progress and uncertainties in attributing the sources of mercury in deposition	Lindberg et al. (2007)
10 (510)	20 (38)	434 (7)	Indigenous knowledge for biodiversity conservation	Gadgil et al. (1993)
11 (501)	287 (7)	43 (25)	The ammonium hypothesis: An additional explanation to the forest dieback in Europe	Nihlgard (1985)
12 (449)	42 (26)	72 (18)	Nitrogen in aquatic ecosystems	Rabalais (2002)
13 (408)	12 (55)	136 (13)	Coupled human and natural systems	Liu et al. (2007)
14 (385)	83 (17)	156 (12)	Reserves, resilience and dynamic landscapes	Bengtsson et al. (2003)
15 (366)	63 (21)	46 (24)	The globalization of nitrogen deposition: Consequences for terrestrial ecosystems	Matson et al. (2002)
16 (363)	287 (7)	197 (11)	Eutrophication and the Baltic Sea: Causes and consequences	Larsson et al. (1985)
17 (322)	417 (5)	12 (44)	Changes in ultraviolet radiation reaching the earth’s surface	Madronich et al. (1995)
18 (312)	15 (49)	156 (12)	The value of coastal wetlands for hurricane protection	Costanza et al. (2008)
19 (307)	175 (10)	43 (25)	How warm was the medieval warm period	Crowley and Lowery (2000)
20 (305)	175 (10)	156 (12)	Nutrient limitation of primary production in the Baltic Sea area	Graneli et al. (1990)

*TC*_2019_, total number of citations from Web of Science Core Collection since publication year to the end of 2019

*C*_2019_, total number of citations from Web of Science Core Collection in 2019

*CN*, *C*_year-1_ + *C*_year-2_; *C*_year-1_, number of citations from JCR year to papers in “year - 1”; *C*_year-2_, number of citations from JCR year to papers in “year - 2”

**Fig. 5:**
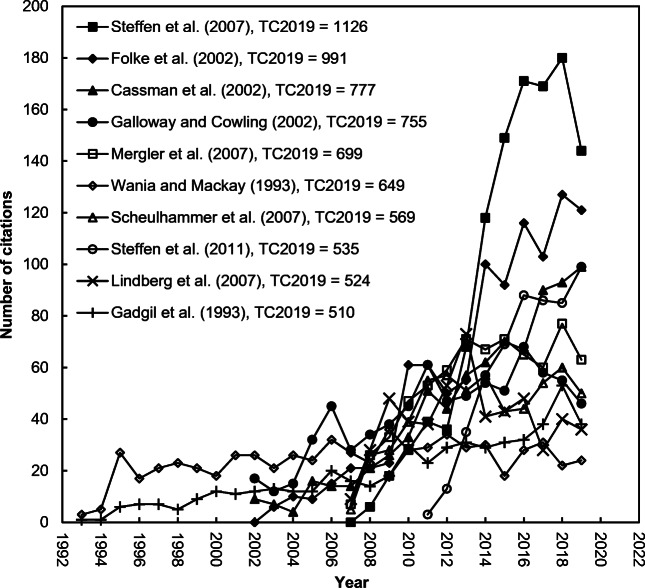
Citation histories of the top ten most frequently cited papers

Citation histories of the top ten most frequently cited papers in *Ambio* are shown in Fig. 5. The classic article by Steffen et al. (2007) had a sharply increased trend after its publication for a decade and then decreased in 2019. Similarly, highly cited articles by Folke et al. (2002) and Steffen et al. (2011) also had an increasing trend. The highly cited article by Wania and Mackay (1993) had an increasing trend after publication for two full years and keep in a plateau. Highly cited papers would not always have a high impact or visibility after publication. Furthermore, only 45 of the top 100 papers in terms of *C*_2019_ were found in the top 100 in terms of *TC*_2019_ in *Ambio*.

### Words in title and author keywords

Several scholars proposed the distribution of words in article titles, abstracts, author keywords, and *KeyWords Plus* in different periods as information to evaluate main research focuses and find their development trends in research topics (Ho and Gatto, 2020). Table 7 showed the top 20 most used words in the paper title. The distribution of the words in four decades was also presented in the table. “Baltic” was the most recurrent word, used in 218 paper titles (6.4% of 3405 papers), followed by management, sea, change, environmental, and climate. “Change” and “climate” were the most popular terms in the last decade. The author’s keywords can be found only in the 2010s in *Ambio*. The top ten used author’s keywords were “climate change,” that can be found in 118 papers (12% of 989 papers in the 2010s), followed by “ecosystem services” (53 papers; 5.4%), “Baltic sea” (47; 5.4%), “eutrophication” (41; 4.1%), “sustainability” (32; 3.2%), “biodiversity” (31; 3.1%), “governance” (31; 3.1%), “phosphorus” (26; 2.6%), “conservation” (25; 2.5%), and “arctic” (23; 2.3%). The results of the analysis considered the author’s keywords and words in the paper title. Papers having the topics of “Baltic sea” and “climate change” were the most popular in *Ambio*, with respective scores of 231 and 345.

**Table 7:** Top 20 most used words in paper title

Words in title	TP	80–19 *R* (%)	80–89 *R* (%)	90–99 *R* (%)	00–09 *R* (%)	10–19 *R* (%)
Baltic	218	1 (6.4)	4 (4.3)	4 (5.3)	1 (7.9)	5 (7.4)
management	216	2 (6.3)	13 (2.8)	1 (6.4)	6 (6.4)	3 (8.4)
sea	204	3 (6.0)	13 (2.8)	8 (4.0)	2 (7.8)	4 (8.3)
change	201	4 (5.9)	269 (0.33)	13 (3.8)	4 (7.1)	1 (10)
environmental	199	5 (5.8)	8 (3.3)	1 (6.4)	5 (7.0)	7 (6.0)
climate	192	6 (5.6)	80 (0.82)	20 (2.9)	3 (7.7)	2 (9.4)
forest	166	7 (4.9)	7 (3.5)	3 (5.6)	9 (5.6)	12 (4.4)
effects	157	8 (4.6)	1 (5.4)	13 (3.8)	8 (5.8)	13 (4.0)
water	156	9 (4.6)	16 (2.6)	17 (3.2)	7 (6.3)	8 (5.7)
development	142	10 (4.2)	1 (5.4)	6 (4.3)	16 (3.7)	20 (3.7)
conservation	140	11 (4.1)	6 (3.8)	10 (3.9)	25 (3.1)	9 (5.4)
ecosystem	133	12 (3.9)	16 (2.6)	41 (1.9)	23 (3.2)	6 (7.1)
Sweden	119	13 (3.5)	12 (3.0)	20 (2.9)	10 (5.3)	32 (2.9)
coastal	118	14 (3.5)	19 (2.0)	15 (3.5)	11 (4.8)	28 (3.2)
case	112	15 (3.3)	115 (0.66)	50 (1.7)	12 (4.7)	10 (5.3)
biodiversity	105	16 (3.1)	N/A	5 (4.9)	38 (2.4)	16 (3.8)
china	105	16 (3.1)	47 (1.2)	8 (4.0)	25 (3.1)	25 (3.4)
global	105	16 (3.1)	13 (2.8)	10 (3.9)	27 (3.0)	36 (2.6)
marine	98	19 (2.9)	11 (3.1)	41 (1.9)	18 (3.6)	29 (3.1)
ecological	95	20 (2.8)	32 (1.5)	23 (2.7)	32 (2.6)	16 (3.8)

*TP*, total number of papers; *R*, rank in a decade; *N/A*, not available

## Conclusion

Research in environmental policy and sustainable development is on the rise. This trend calls for accurate mapping of existing publications in these domains (Gatto et al. 2021; Farrukh et al. 2020). A large number of bibliometrics have been focusing on deducting and examining the essential features and trends for specific journals’ research products (Kumar et al. 2020a, b; Arkhipov 1999). *Ambio* is a top journal in environmental sciences and policy and sustainability studies. Several investigations have conducted bibliometric analyses on journals’ publications performances over the years. These facts motivated this paper’s rationale in scrutinizing the main publication features of *Ambio* for the time span 1980–2019. This time frame is the available indexing period for the journal and refer to the Web of Science categories of environmental sciences and environmental engineering—the two original categories for which the journal has been historically indexed.

A total of 3405 papers including 3263 articles and 142 reviews in *Ambio* from 1980 to 2019 was analyzed. The peak year of citations per publication was found to be the 3rd full year since its publication. The papers published in 2007 and 2002 had a higher impact on the journal. Sweden dominated the performance indicators, ranking first in six publication indicators. Stockholm University and the Swedish University of Agricultural Sciences in Sweden were the best performers. T.V. Callaghan did not only publish the largest number of papers in *Ambio* but was also the best performer for the categories of first-author paper and corresponding-author papers. The less productive author, J. Rockstrom was, however, the largest journal impact factor contributor.

One-third of the top 100 *IF* contributing papers were found in the top 100 highly cited papers. Only 45% of the top 100 most impactful papers in 2019 were listed within the top 100 highly cited papers. Three members of the advisory board in the *Ambio*—T.V. Callaghan, C. Folke, and T.D. Prowse—were ranked as productive authors. One article published by Rockstrom et al. in 2017 was the top journal impact factor contributing article. An article by Steffen et al. in 2007 was not only the most frequently cited article but also the most impactful publication in 2019. The results show that the journal impact factor contributing papers were not the highly cited papers or the highly impactful publications in *Ambio* in the most recent available year—2019.

The results in the study provided some initial evidence that highly cited papers in a journal might not necessarily greatly contribute to the journal impact factor. This observation leads to a striking conclusion: it is not always appropriate to use the journal impact factor as an indicator to evaluate the publication performance of institutes and authors. This conclusion may lead to important research, policy, and practical implications in the field of bibliometrics, scientometrics, information science, and publications studies that may be carried on in other papers.

This work also attempted to provide some technical and analytical innovations with respect to the existing scholarship. Some of the novelties proposed in this study are the procedural improvements performed with the scope to render more solid and reliable outputs and avoid overlappings and errors. Additionally, the paper benefited from an enlarged set of new characteristics—that has been investigated along with traditional publication features. These distinct elements allowed for increased precision in the analyses and for broadening the investigation spectrum, returning more accountable results.

Bibliometric analyses can offer a bird’s eye perspective on specific timely subjects. This includes specific journals’ characteristics and publications trends, as well as quantitative indications on the analyzed literature. However, bibliometrics is unable to yield recommendations on germane publications features, including papers’ contents, indications and quality. This is why bibliometrics suggests for integration of qualitative studies on the same bodies of research and mixed methods. In this regard, the paper at hand makes no exception. Future papers may address this issue, disentangling selected characteristics of the examined works. Furthermore, other studies may target different environmental policy and science and sustainability studies journals, as well as further disciplines. Alternative bibliometric techniques, publications databases, papers characteristics, software, and time frames could also be considered. Differentiating the data and the analyses would contribute to better assess the trends and determinants of the sorting sectoral publications.

## Footnotes

1 https://support.clarivate.com/ScientificandAcademicResearch/s/article/Journal-Citation-Reports-Document-Types-Included-in-the-Impact-Factor-Calculation?language= en_US.

2 http://ipscience-help.thomsonreuters.com/inCites2Live/indicatorsGroup/aboutHandbook/usingCitationIndicatorsWisely/jif.html
